# Supplementary material for: Healing Bodies, Healing Communities: A Community-Based Qualitative Study of Adult Survivors of Childhood Sexual Trauma in South Africa
Source: Healthcare (Basel). 2025 Oct 15;13(20):2601. doi: 10.3390/healthcare13202601 (PMC12563590; doi:10.3390/healthcare13202601)
Supplement: Supplementary file 1 [file healthcare-13-02601-s001.zip › healthcare-3869473-supplementary.pdf]

Table S1. Summarized individual participant care pathway co-development processes.

Participant	Session	Phase	Theme	Affective valence	Self perception	Therapist response	Timestamp	Timeline
Sarah	1	3	Black sheep	x	x	Non-judgement	00:01:15	
	1	3	Darkness	x	x	Non-Pathologising	00:09:11	
	1	3	Façade	x	x	Acknowledge	00:10:03	
	1	3	Suicidal	x	x	Connect to pain	00:17:16	
	1	5	Pain/Need Safety	x	Chest/Heart	Hold Space - Embodied	01:03:12	
	1	7	Trust/ Need Safety	x	x	Reframe/Rapport	01:05:10	
	1	3	Loneliness	✓	Chest/Heart	Sadness/Isolation	00:04:24	
	2	3	Turmoil/Façade	x	x	Hold Space - Fear	00:32:33	01:38:05
	3	5	Sadness	✓	Chest/Heart	Hold Space – Emotion	00:03:11	02:07:35
	4	8	Empowered/Peace	✓	✓	Authenticity Reframe	00:01:48	03:18:22
	4	8	Authentic Emotion	✓	✓	Acknowledge	00:02:08	03:18:42
	5	4	Suppression/Facade	x	✓	Identify Coping Style	00:03:21	04:07:40
	5	4-8	Integration	x	x	Revisit Theme	00:38:15	04:42:34
	5	8	Integration	x	Chest/Heart	Hold Space - Embodied	00:44:40	04:48:59
	6	8	Freedom/Peace	✓	✓	Reflect Back/New Theme	00:13:16	05:06:54
	6	8	Liberative	✓	✓	Authentic/Empowered	00:03:24	05:10:18
Mia	1	3,5	Sexual Objectification	✓	x	Allow Emotion	00:01:15	
	1	3	Darkness	x	x	Religious Framing	00:23:04	
	1	4,6	Anger	✓	x	Allow Emotion	00:25:37	
	2	5,6	Intense Fear	✓	Legs/Chest	Visceral Memory	00:11:53	01:13:45
	2	5,6,8	Intense Fear	✓	Whole Body	Hold Space - Emotion	00:17:05	01:19:57
	2	5,6,8	Allow Rest	✓	✓	Rest/Integration	00:54:55	01:57:47
	2	5,6	Fear/Exhaustion	✓	Legs/Chest	Trauma Response	00:55:02	01:57:54

	3	3,5,6,8	Fear	✓	✓	Integration	00:02:05	01:59:23
	3	3,5,6,8	Hatred	✓	x	Hold Space - Emotion	00:04:15	02:01:33
	4	4	Unemotional	x	✓	Numbness	00:00:15	02:31:09
	4	5,6,8	Embodied Release	✓	Chest	Integration	00:05:16	02:36:10
	5	5-7	Social Anxiety	✓	✓	Integration	00:06:23	02:58:10
	6	5,6,8	Selfcare	✓	Whole Body	Integration	00:00:10	03:25:22
	8	4-8	Energized/Positive	✓	✓	11 Month*FU		
Ava	1	2	Open ended Q*	x	x	Spontaneous *TD	00:00:15	
	1	3	Incest/Rape	x	Heart	Embodied Dissociation	00:00:49	
	1	3	Gangrape	x	x	Dissociation	00:03:04	
	1	4	Emotional Numbness	x	x	Dissociation	00:03:50	
	1	3	Anxiety/Fear/Numbness	✓	x	Hold Space - Emotion	00:19:20	00:23:33
	2	4,5	Loss of Appetite/Sleep	x	✓	Trauma Response	00:00:15	00:23:48
	2	4	Emotionally Dead	x	✓	Dissociation	00:03:44	00:27:44
	2	6,7	Vulnerable	✓	x	Hold Space - Emotion	00:04:07	00:29:07
	2	3,4	Memory Flood	✓	x	Holding Space	00:14:50	00:39:18
	3	4,5	Pain/Fear	✓	Stomach	Holding Space - Emotion	00:10:54	01:18:00
	3	4,5	Numbness - Body	x	Heart	Awareness - Embodied	00:11:49	01:29:49
	7,8	4-8	Voicing needs/Appetite/ Energized	✓	✓	Integration	00:12:24	03:21:04
Charlotte	1	2,3	Open ended	x	x	Spontaneous Disclosure	00:12:30	
	1	4	Therapeutic/ Co-dependent	x	x	8years DBT	00:14:30	
	1	3	Mistrust	x	x	Trauma Response	00:18:55	
	1	3,4	Need for safety	x	x	Holding Space	00:14:30	
	1	3,4	Anger	✓	x	Holding Space - Emotion	00:25:38	

	1	7	Therapeutic Framing	✓	✓	Anger – Allow Pain	00:26:42	
	1	3,4	Abuse Disclosure	x	x	Dissociation	00:33:30	00:50:09
	2	4-6	Fear	✓	✓	Hold Space - Embodied	00:01:20	00:51:29
	2	4-6	Fear/Alone/Broken	✓	✓	Hold Space - Authentic	00:26:23	01:15:12
	2	3-6	Memories Accessed	✓	✓	Hold Space – Emotions	00:29:58	01:18:47
	2	3-6	Tired/Relief	✓	✓	Trauma Integration	00:32:33	01:21:22
Joan	1	2	Open-ended	x	x	Spontaneous Disclosure	00:00:09	
	1	3	Religious Theme/ Stumbling Block	x	x	Holding Space	00:00:43	
	1	3	Trauma Disclosure	✓	x	Holding Space - Emotion	00:02:18	00:03:14
	1	3-6	Relief	✓	x	Authenticity	00:06:32	00:07:28
	1	4	Fear/Dissociation	✓	x	Auditory Trauma Memory	00:09:07	
	1	4	Isolation/Trauma Suppression	x	x	Hold Space -Shame	00:01:59	
	2	4,6	Visual Memory	✓	x	Hold Space - Emotions	00:00:22	00:39:10
	2	4	Visceral Shaking	✓	✓	Hold Space - Body	00:13:34	00:52:32
	2	2-4,7	Embodied Awareness	✓	✓	Integration	00:26:11	01:05:09
	2	4	Intense Fear	✓	✓	Integration	00:28:23	01:07:21
	2	4	Religious Coping	x	x	Dissociation	00:36:40	01:15:38
	3	4-6	Sadness	✓	x	Hold Space - Emotions	00:19:51	01:36:51
	3	4-6	Calmer	✓	✓	Integration	00:26:58	01:43:58
	4	4-6	Fear/Dissociation	✓	x	Religious Copings Style	00:11:09	02:05:06
Lydia	1	3	Open ended	x	x	Spontaneous Disclosure	00:01:14	
	1	3	Dissociation/Shame	x	x	Hold Space	00:02:15	
	1	3,5	Trauma Disclosure	x	x	Hold Space	00:03:58	00:07:27

	1	2,7	Sadness/Bitterness	✓	Heart	Embodied Awareness	00:13:12	00:16:42
	1	4	Religious Dissociation/Guilt	x	x	Coping Style	00:34:02	00:38:48
	2	4	Guilt	x	x	Hold Space	00:36:12	
	2	4-6,8	Release	✓	✓	Embodied Awareness	00:28:19	
	4	4-6	Tired/Relieved	✓	✓	Integration	00:09:40	
Evelyn	1	2,3	Open ended	x	x	Spontaneous Disclosure	00:00:11	
	1	2,7	Burdened	x	x	Trauma Response	00:00:30	
	1	3,4	Constant Fear/Barrier	✓	Stomach Pain	Hold Space - Body	00:04:13	00:04:54
	1	4,7	Fear	✓	x	Hold Space - Emotions	00:04:13	
	1	4,6	Detailed Trauma Disclosure	✓	x	Embodied Awareness	00:51:20	01:12:30
	2	7	Burdened	x	Stomach Pain	Hold Space - Body	00:24:47	01:37:17
	2	4-7	Sadness/Memory	✓	x	Hold Space - Emotions	00:30:20	
	2	4-6	Cold/Bladder	✓	✓	Integration	01:00:46	
	3	5,6	Interpersonal Boundaries	✓	x	Affective Awareness	00:03:55	
	5	4,5,7	Fear	✓	x	Hold Space - Emotions	00:34:25	
	5	5-7	Sadness	✓	✓	Integration	01:20:00	
Aisha	1	2,3	Religion/Forgiveness	x	x	Coping Style	Process Notes	
	2	3	Tired/Self-hatred	✓	x	Hold Space - Emotions	Process Notes	
	3	4-6	Severe Anger	✓	Whole Body	Appropriate Emotion	Process Notes	02:27:55
	4	4-6	Rejection	✓	x	Affective Awareness	00:14:32	02:42:28
	4	4-6	Fear/Coldness	✓	✓	Integration	00:22:31	02:50:29
	5	4-6	Anger	✓	x	Affective Awareness	00:01:51	03:23:28
	7	5,6,8	No fear/Peace	✓	✓	Integration	00:08:45	04:23:22
Amber	1	3	Open ended	✓	x	Spontaneous Disclosure	00:01:09	

	1	3	Persistent Sadness	✓	x	Affective Awareness	00:01:55	00:01:55
	1	3	Rape Memory	x	x	Holding Space	00:02:11	00:02:11
	1	4-6	Sadness	✓	Chest/Lungs	Holding Space - Embodied	00:43:46	01:01:23
	2	4-6	At Peace	✓	x	Holding Space – Emotions	00:11:57	01:13:20
	4	4-7	Fear	x	Tight Chest	Holding Space – Embodied	00:19:50	02:55:35
	4	4-7	At Peace	✓	✓	Integration	00:41:13	
	6	5-7	Physical Safety	✓	x	Family Stressors	00:03:37	
Mandy	1	3	Open ended	✓	x	Spontaneous Disclosure	00:14:54	
	1	3,4,6	Severe Consistent Sexual Abuse	✓	x	Affective Awareness	00:22:10	00:39:07
	2	4,6	Sadness	✓	x	Appropriate Sadness	00:08:23	00:47:30
	2	4,6,7	Isolation	✓	x	Trauma Response	00:09:15	
	2	4-6	Sadness	✓	Heart	Embodied Awareness	00:12:49	00:51:56
	3	4	Chooses to not revisit memories	x	x	Process Consent	00:01:29	
	4	4-7	Tiredness	✓	Lower back	Hold Space - Embodiment	00:10:32	
Debra	1	3	Open ended	✓	x	Spontaneous disclosure	00:02:39	
	1	3,5	Rape Disclosure	✓	x	Embodied Awareness	00:02:50	
	1	3,6	Anger	✓	x	Hold Space – Appropriate Anger	00:10:58	
	1	4	Suicidal Ideation	x	✓	Affective Awareness	00:11:33	
	1	3,4	Fear	✓	x	Holding Space – Emotions	00:05:30	
	1	4-6	Memory accessed	x	✓	Embodied Awareness	00:36:45	
	1	4-7	Visceral Trauma Memory	✓	✓	Integration	00:04:21	00:46:40
	3	4-6	Emotional Turmoil	✓	✓	Integration	00:12:14	01:58:02
	4	4-6	Fear	✓	x	Holding Space - Allow Emotion	00:16:14	02:57:40

	4	7	Memory Accessed	x	✓	Integration	00:31:12	03:12:38
	4	7	Peaceful	✓	✓	Integration	00:42:06	03:23:30
Zara	1	3	Open ended	x	x	Disclosure	00:00:28	
	1	4	Perpetrator named	✓	x	Authenticity	00:09:13	00:52:41
	2	7	Vulnerability	✓	x	Holding Space	00:07:07	00:59:48
	2	3,4	Culture/Non-disclosure	x	x	Systemic Abuse	00:09:17	01:11:44
	2	7	Religious Intervention	x	x	Disembodied/ Cognitive	00:45:32	
	3	4-6	Emotional Suppression	✓	Upper body	Embodied Awareness	00:01:46	01:53:25
	3	4-6	Performative	x	x	Coping Style	00:08:05	
	3	4-6	Anger towards God	✓	x	Hold Space – Emotion	00:08:17	01:59:56
	4	4-6	Sadness	✓	Chest	Integration	00:04:26	03:14:45
	4	4	Performative	x	x	Coping Style	00:49:49	
Violet	1	2	Open ended	x	x	Spontaneous	00:00:10	
	1	3,4	Fibromyalgia / Sadness	x	x	Dissociation	00:01:09	
	1	4	Religious Coping style	x	x	Theme	00:04:23	
	1	3	Rape Disclosure	✓	✓	Authenticity	01:12:12	
	1	3-6	Trauma Memory	✓	✓	Holding Space Embodied	00:08:31	00:54:33
	2	3-6	Anger	✓	Feet/Lower Body	Holding Space	00:26:33	01:38:54
	2	7	Relief/Peace	✓	✓	Integration	01:01:31	01:43:57
	5	4-6	Tiredness	✓	✓	Integration	00:38:11	04:43:58
	5	3-6	Appropriate Anger	✓	✓	Integration	00:41:43	04:47:30
	5	4-6	Visceral Memory	✓	Nausea	Integration	00:45:10	04:50:57
	6	7	Selfcare	✓	✓	Integration	00:00:20	05:24:53
	8	8	Energized/ No muscle spasms	✓	✓	7 Month Follow-up		

DBT=Dialectic Behavioural Therapy; **TD**=Trauma Disclosure; **Q**=Questions**;** **TD**=Trauma disclosure; **FU**=Follow-up. **Affective valence/Self-perception**=The table includes an indication of whether the affective valence and/or; embodied awareness was present **(✓)**, or not **(X),** providing insight into how the participant's trauma awareness and integration evolved.
